# Characteristics of the regulation of the surprise emotion

**DOI:** 10.1038/s41598-019-42951-y

**Published:** 2019-05-20

**Authors:** Chuanlin Zhu, Ping Li, Zhao Zhang, Dianzhi Liu, Wenbo Luo

**Affiliations:** 1 School of Education, Soochow University, Suzhou, 215123 China; 2 Research Center of Brain and Cognitive Neuroscience, Liaoning Normal University, Dalian, 116029 China; 3 Laboratory of Emotion and Mental Health, Chongqing University of Arts and Sciences, Chongqing, 402168 China

**Keywords:** Cognitive neuroscience, Human behaviour

## Abstract

This study aimed to assess the characteristics of the regulation of the emotion of surprise. Event-related potentials (ERPs) of college students when using cognitive reappraisal and expressive suppression to regulate their surprise level were recorded. Different contexts were presented to participants, followed by the image of surprised facial expression; subsequently, using a 9-point scale, participants were asked to rate the intensity of their emotional experience. The behavioral results suggest that individuals’ surprise level could be reduced by using both expressive suppression and cognitive reappraisal, in basic and complex conditions. The ERP results showed that (1) the N170 amplitudes were larger in complex than basic contexts, and those elicited by using expressive suppression and cognitive reappraisal showed no significant differences, suggesting that emotion regulation did not occur at this stage; (2) the LPC amplitudes elicited by using cognitive reappraisal and expressive suppression were smaller than those elicited by free viewing in both context conditions, suggesting that the late stage of individuals’ processing of surprised faces was influenced by emotion regulation. This study found that conscious emotional regulation occurred in the late stages when individuals processed surprise, and the regulation effects of cognitive reappraisal and expressive suppression were equivalent.

## Introduction

When we pass an important test, some of us dance out of excitement; when we are informed about the sudden death of a loved one, we feel extremely sad. The “excitement” and “sadness” are different emotional experiences. Emotion is a complex psychological phenomenon, involving an individual’s subjective experience, external behavior (for example, facial expressions), and physiological responses^1,2^. The same emotional experience may have positive and negative consequences for individuals; when the negative consequences weigh more than the positive ones, the emotion needs to be regulated. In certain situations, we need to regulate our emotions, for example, images of certain humorous movies may be imprinted in your mind, but you may need to stifle your laughter when attending a funeral. Emotion regulation refers to individuals’ attempts to influence the emotions that they experience, when they have them, and how they are experienced and expressed^3,4^.

Researchers have conducted a series of in-depth studies regarding emotion regulation and obtained fruitful results. For example, according to Gross^3,5^, emotion generation is a dynamic process, and each of its stages can become the object of regulation; thus, the process of emotion regulation is also dynamic, based on which he put forward the process model of emotion regulation. This model includes situation selection, situation modification, attention deployment, cognitive change, and response modulation. The first four strategies emerge before the specified emotional reaction, and are thus known as antecedent-focused emotion regulation strategies, while response modulation is a response-focused emotion regulation strategy, since it emerges after the specified emotional reaction. Cognitive reappraisal and expressive suppression are specific strategies representing the two types of emotion regulation strategies, respectively^6^. Cognitive reappraisal refers to cognitive changes, including changing ones’ understanding of the emotional events and the influence of these emotional events on oneself and others. This strategy leads individuals to appraise emotional events, especially negative emotions, in a more positive way, thereby reducing their negative impact. Expressive suppression belongs to response modulation and refers to the inhibition of the expression of ongoing or potential emotions. Expressive suppression can activate one’s self-control process to suppress emotional behavior.

Many studies have been conducted regarding cognitive reappraisal and expressive suppression, which have been compared using emotions with a valence, such as anger^7^, sadness^8,9^, frustration^10^, and fear^11^, as the target emotion to be regulated. Generally, participants could process and regulate these emotions successfully, despite lacking contextual information. For example, in a previous study^11^, participants employed either expressive suppression or cognitive reappraisal to regulate their experience of fear or disgust during video exposure. The results showed that participants’ emotional distress showed no significant difference between the two strategies when watching fear-related films. However, significantly less emotional distress was experienced when using cognitive reappraisal compared with expressive suppression when watching disgust-related films, which suggests that cognitive reappraisal is more effective than expressive suppression when regulating disgust. Similarly, another study^7^ showed that cognitive reappraisal was more effective in reducing anger than was expressive suppression.

On the other hand, individuals often experience emotions without a specific valence, for example, surprise. In order to process surprise more effectively, individuals need to make full use of relevant background information^12–15^. In a study by Kim *et al*.^12^, participants viewed a surprised face, which was preceded by a sentence with either a positive or negative valence (e.g., She just lost $500 vs. She just found $500). Their results showed that positively-cued surprised faces produced lower ventral amygdala activation than did negatively-cued surprised faces. Additionally, other researchers^15^ found significant differences in the activation of the amygdala by positive and negative surprise, specifically, there was heightened amygdala activity in response to positive (vs. negative) surprise. Some reviews^16,17^ revealed that the effects of emotion regulation are influenced by external environmental (contextual) factors. However, as far as we know, no studies have investigated the characteristics of the regulation of surprise preceded by different contexts. Hence, what features will emerge when individuals use different strategies to regulate surprise that is cued by different contexts?

Researchers have conducted numerous ERP studies on emotional facial expression processing and emotion regulation. Two representative ERP components have been investigated in these studies, namely N170 and late positive complex (LPC). The N170 is an early negative-going potential over occipito-temporal regions usually peaking at around 170 ms post-stimulus^18,19^, and is considered to be an indicator of automatic or unconscious processing of facial expressions^20,21^. Numerous previous studies showed that the N170 ERP component was particularly sensitive to faces, that is, the N170 amplitudes elicited by facial expressions were larger than those elicited by non-facial stimuli^22–24^. Additionally, some researchers found the N170 amplitudes elicited by viewing faces of other races (vs. own-race) were larger^25–27^, which suggests that the N170 may represent social categorization processes.

The other well-known ERP component for measuring processing of emotional information is the LPC. This is a centro-parietal positive-going wave, which peaks typically at 400–700 ms after stimulus onset^28,29^. Importantly, some studies indicated that the LPC amplitude was sensitive to emotional intensity and the evaluation operation that changes the meaning conveyed by emotional stimuli. For example, in a study by Hajcak and Nieuwenhuis^30^, participants were asked to employ a specified emotion regulation strategy (cognitive reappraisal or free viewing), while watching emotional (positive, neutral, and negative) pictures. The results showed that the LPC amplitudes decreased when using cognitive reappraisal compared with free viewing. Additionally, the reduction in self-reported emotional intensity after using the specified emotion regulation strategy was positively related to the degree of LPC modulation. Soon after that, Thiruchselvam *et al*.^31^ conducted a similar experiment, which showed that both distraction and cognitive reappraisal reduced the LPC amplitudes in comparison with free viewing, but the LPC amplitudes decreased earlier when using distraction (vs. cognitive reappraisal). These findings were supported by subsequent studies^32,33^. Moreover, it has been suggested that the LPC amplitude is an effective index of emotion regulation^34–36^.

Based on the information above, this study aimed to explore the characteristics of the regulation of surprise. Considering the vital role of N170 in processing emotional facial expressions^23,37,38^, and the role of LPC in emotion processing^32,34,36^, we hypothesized that the N170 amplitudes would be influenced by the type of context, but would not be affected by the type of emotional regulation strategy (Hypothesis 1); and the LPC amplitudes would be influenced by the type of emotion regulation strategy: those elicited by using cognitive reappraisal would not always be smaller than those elicited by using expressive suppression, to be more specific, the LPC amplitudes elicited by the two strategies would be equivalent, or the LPC amplitudes elicited by using expressive suppression (vs. cognitive reappraisal) would be smaller (Hypothesis 2).

## Results

### Behavioral Results

#### Manipulation check

As described in the Methods parts, at the end of each block, participants were asked to report their proficiency at using different strategies on a 9-point scale (1 = not skilled, 9 = very skilled). The results of one-sample t-test for task 2 showed that the scores of proficiency (6.864 ± 1.504, M ± SD) were significantly higher than chance level (i.e., the midpoint of the 9-point scale, 5), *t* = 14.667, *df* = 139, *p* < 0.001, suggesting that participants successfully used the specified strategies.

#### The assessment of emotional experience intensity

The results of 2 (context) × 3 (emotion regulation strategy) repeated-measures AVOVA showed that the main effect of emotion regulation strategy type was significant, *F* (2, 54) = 24.567, *p* < 0.001, *η*^2^ = 0.476, the post-hoc analysis showed that the emotional experience intensity after using cognitive reappraisal (M ± SD, 3.950 ± 0.220) and expressive suppression (3.713 ± 0.217) were lower(*p*s < 0.001) than those using freely view (4.326 ± 0.176, *p* < 0.001), while using the cognitive reappraisal and expressive suppression showed no significant difference (*p* > 0.05). The main effect of context type and the interaction of emotion regulation strategy type with context type were not significant.

#### ERP results

**Figure 1:**
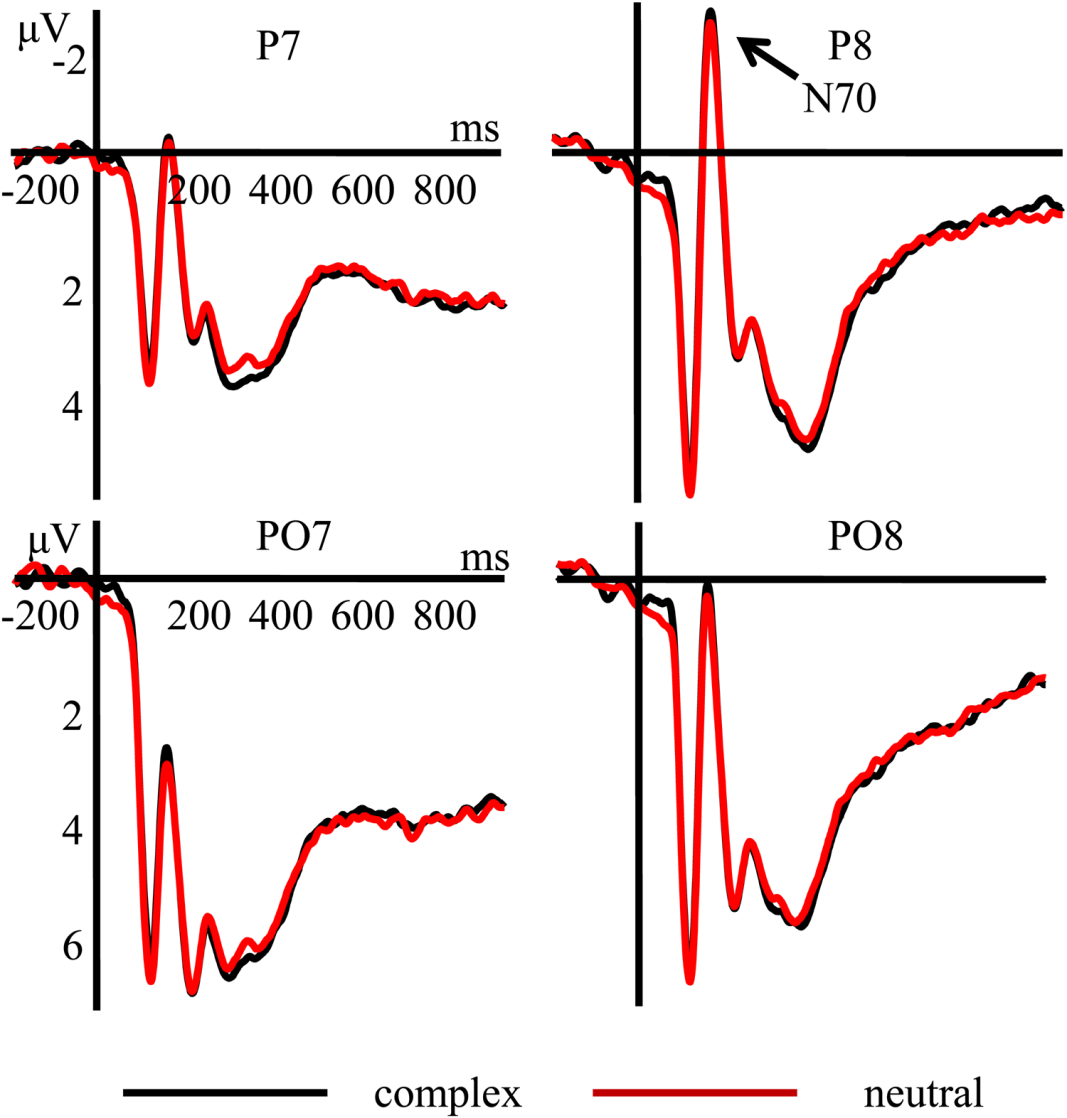
Grand average ERPs of N170 component in the basic and complex conditions recorded at P7, P8, PO7, PO8.

N170 The main effect of context was significant, *F* (1, 27) = 6.014, *p* = 0.021, *η*^2^ = 0.182, the post-hoc analysis showed that the N170 amplitudes under the complex condition (M ± SD, 4.992 ± 0.708 μV) were larger (*p* = 0.021) than those under the basic condition(5.208 ± 0.711 μV) (Fig. 1). The main effect of electrode was significant, *F* (3, 81) = 4.611, *p* = 0.013, *η*^2^ = 0.146, the post-hoc analysis showed that the N170 amplitudes at the PO7 (6.227 ± 0.850 μV) were larger (*p* = 0.001) than those at P7 (3.635 ± 0.624 μV). No other significant main effect or interaction effect was found for N170 amplitudes.

LPC To test the points at which each regulation strategy modulated the LPC, the LPC (400–1000 ms) was divided into three equal 200-ms time segments (400–600, 600–800, and 800–1000 ms).

**Figure 2:**
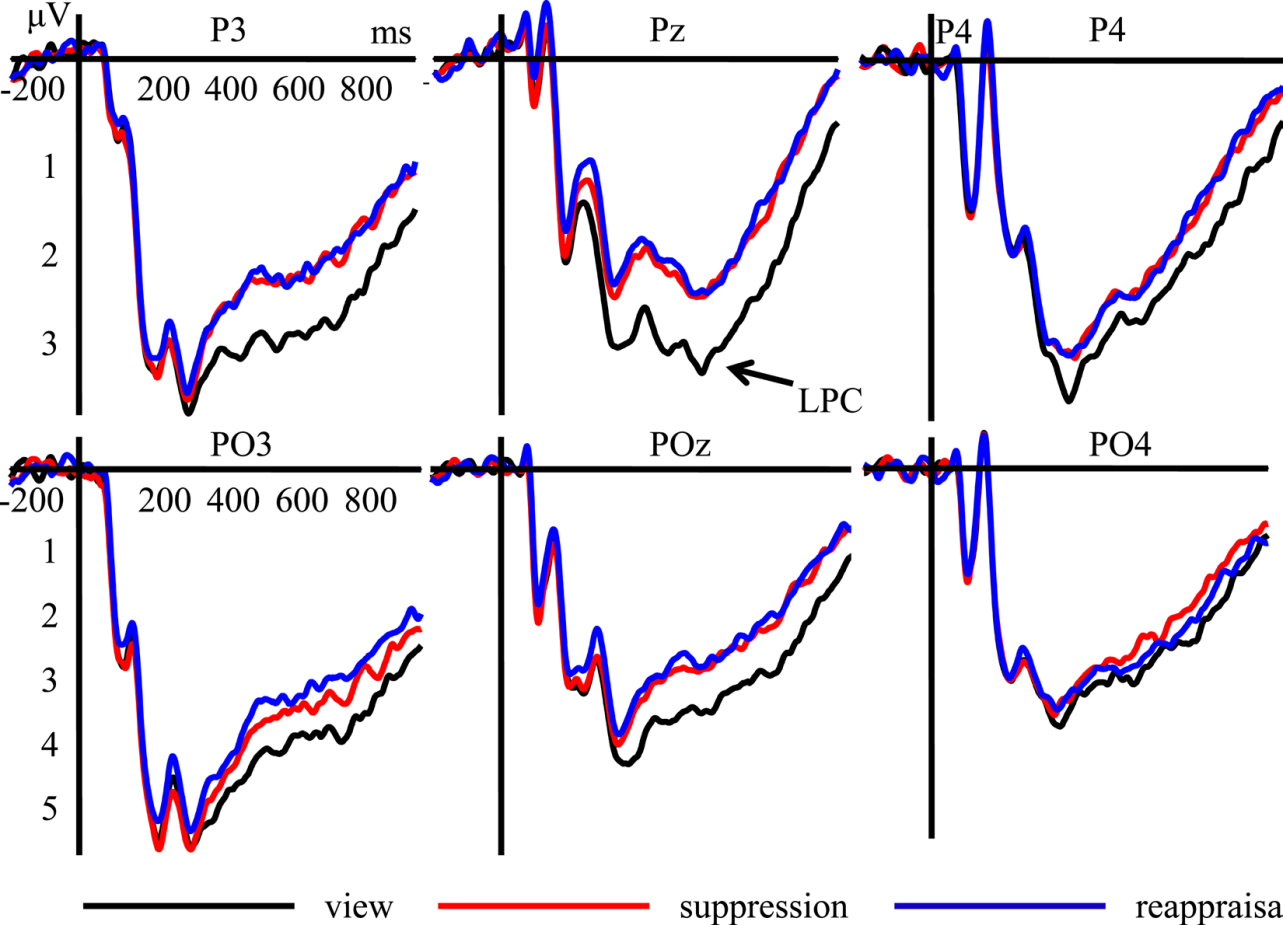
Grand average ERPs of LPC component of participants using different emotion regulation strategies recorded at P3, P4, Pz, PO3, PO4, and POz.

400–600 ms The main effect of emotion regulation strategies was significant, *F*(2,54) = 8.337, *p* = 0.001, *η*^2^ = 0.236. The amplitudes of the LPC induced by using expressive suppression (3.529 ± 0.345 μV, *p* = 0.008) and cognitive reappraisal (3.515 ± 0.310 μV, p = 0.006) were smaller than those induced by using free viewing (3.973 ± 0.396 μV), while the LPC amplitudes elicited by using expressive suppression and cognitive reappraisal showed no significant difference (Fig. 2). The main effect of electrode was significant, *F*(5, 135) = 5.709, *p* = 0.004, *η*^2^ = 0.175, and the post-hoc analysis showed that the LPC amplitudes were largest at the PO3 electrode (4.617 ± 0.575 μV). No other significant main or interaction effects were found for LPC amplitudes.

600–800 ms The main effect of electrode was significant, *F*(5, 135) = 5.391, *p* = 0.005, *η*^2^ = 0.166, and the post-hoc analysis showed that larger LPC amplitudes were elicited at the PO3 electrode (4.336 ± 0.537 μV) than at the other electrodes. No other significant main or interaction effects were found for LPC amplitudes.

800–1000 ms The main effect of electrode was significant, *F*(5, 135) = 5.656, *p* = 0.002, *η*^2^ = 0.173, and the post-hoc analysis showed that the LPC amplitudes at the PO3 electrode (3.677 ± 0.496 μV) were larger than at the other electrodes. No other significant main or interaction effects were found for LPC amplitudes.

**Figure 3:**
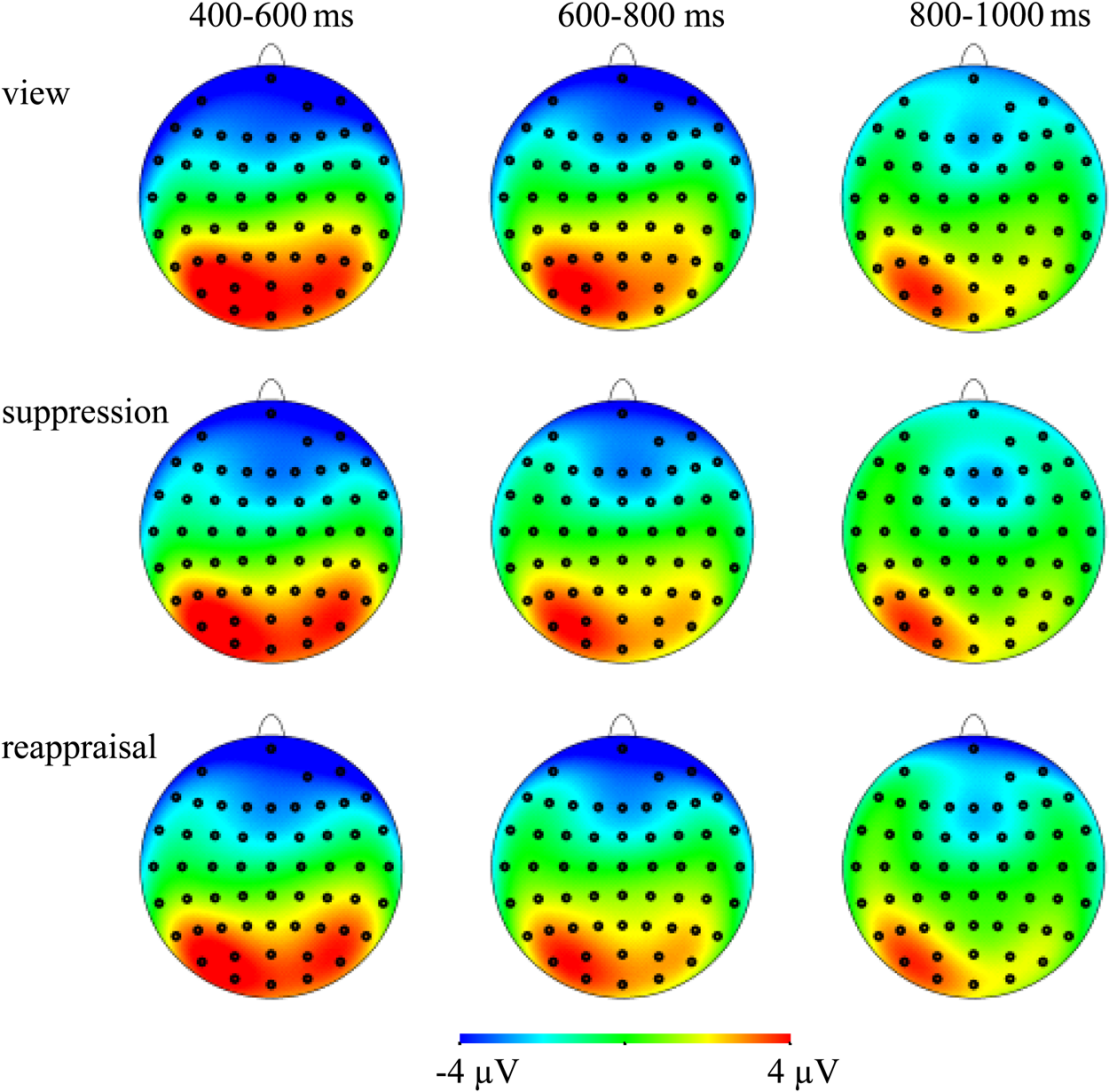
Average ERP topographies of LPC component elicited by using free viewing, expressive suppression, and cognitive reappraisal in three time windows.

The ERP topographies of the LPC elicited by different emotion regulation strategies in different time windows are shown in Fig. 3.

## Discussion

The characteristics of the regulation of emotions with a certain valence have been widely studied, unlike those of emotions without a specific valence, such as surprise. Adopting the ERP technique and choosing surprise as the target emotion, the present study is the first to explore the characteristics of the regulation of surprise in different contexts with a group of college students. The behavioral data indicated that all participants were skilled in the use of specific strategies to regulate their emotions, and the intensity of the emotional experience after using expressive suppression and cognitive reappraisal (no difference) was lower than that after using free viewing, verifying that the regulation effect of expressive suppression and cognitive reappraisal showed no significant difference, which was consistent with the findings of previous studies^39,40^.

The ERP results showed that the N170 amplitudes in the complex condition were larger than in the neutral condition; thus, Hypothesis 1 was confirmed. The facial expressions in the complex condition involved happy and sad features, with the eyes and mouth being more striking compared to those in the neutral condition. Numerous previous studies indicated that the N170 is involved in the configural processing of visual facial features^41–43^, and the N170 amplitudes are enhanced when faces are attended to^44^; thus, it is reasonable to speculate that the larger N170 amplitudes in the present study were due to more attention resources being allocated to the complex (vs. neutral) images. Additionally, the N170 amplitudes elicited by using different emotion regulation strategies showed no significant differences, suggesting that they were not influenced by the type of emotion regulation strategy. In a recent study^45^, participants were shown negative emotional cue words, presented prior to happy or fearful faces. Two emotion regulation strategies were used: participants were asked to down-regulate their emotions to each of the faces by using the negated emotional cue words, or just attend to the faces without active regulation. The results showed that the N170 amplitudes elicited by using the two emotion regulation strategies showed no significant differences, which is in line with the results of the present study to some extent. The reason for this may be that the N170 component occurs in an early stage when the conscious cognitive processing has not yet begun^46^.

The LPC amplitudes elicited by free viewing were larger than those elicited by cognitive reappraisal and expressive suppression, while the corresponding amplitudes elicited by these latter two strategies showed no significant difference, both in complex and basic situations. Thus, the results of the present study suggest that the regulation effects of expressive suppression and cognitive reappraisal were equivalent when processing surprise, both in complex and basic situations. In a previous study^39^, participants used expressive suppression or cognitive reappraisal while watching positive video clips. Subsequently, participants judged the valence and arousal level of the film, and the results showed that cognitive reappraisal (vs. expressive suppression) led to less positive valence ratings, but the arousal level after using cognitive reappraisal and expressive suppression showed no significant difference. Similar results have been found in other studies^11,40^, which are in agreement with our results.

However, the present results do not support the cognitive reappraisal advantage effect mentioned in the Introduction. This contradiction may be explained by the following factors. To begin with, the influence of contexts in our study differs from that in other research. In previous studies, the target emotions included anger^7^, anxiety^47^, and sadness^9^, all of which have a certain valence (negative), allowing the participants to regulate emotions smoothly. Thus, the effect of the context in emotion regulation may not be as important in such studies. However, in this study, the target emotion (surprise) does not have a specific valence. Consequently, if participants want to process and regulate their emotions successfully, they need to rely on contextual information; in this study, we did in fact provide different contexts, and this is one of the innovations of our research. In addition, the potential influence of the differences between Eastern and Western cultures could also help explain our findings. Although both the present and previous studies included college students as participants, in the present and many other studies^48,49^, participants were Chinese students who hold oriental cultural values, whereas participants in other studies^9^ may have held Western cultural values. Numerous studies^48,50,51^ have indicated that compared with individuals holding Western cultural values, those with Eastern cultural values are more worried that their improper behavior may harm interpersonal relationships, and more inclined to suppress their emotions, especially negative feelings, in different situations.

In a previous study^52^, participants were shown a series of stimuli consisting of voice cues (presented prior to each of the target stimuli) and emotional scenes (target stimuli from IAPS), and rated how they felt during the target stimuli presentation using the Self-Assessment Manikins scales^53^. A total of five experimental conditions were used: neutral (cue)-neutral (target emotion), neutral-negative, neutral-positive, unpleasant-negative, and pleasant-positive. The results showed that participants were more inclined to judge the target emotion as negative, and their negative emotional experiences were more intense in the “unpleasant-negative” (vs. “neutral-negative”) condition. Meanwhile, participants were more inclined to judge the target emotion as positive in the “pleasant-positive” (vs. “neutral-positive”) condition. Similarly, in a latter study^36^, healthy individuals and schizophrenia patients were presented with negative or neutral pictures (target stimuli), preceded by an audio description (appraisal frames) of the target stimuli as being either negative or neutral, and then rated how negative the picture made them feel. The results showed that the LPC amplitudes elicited by the negative pictures preceded by a neutral (vs. negative) appraisal frame were smaller. These studies indicate that the role of context should be considered when studying individuals’ emotional experience and regulation. Thus, it is reasonable to consider the potential influence of context on the regulatory effects of different strategies in the present study.

The limitations of the present study and the directions for future study are as follows. First, the images used to create complex situations were produced using FaceMorpher Lite 2.5 software, and it should be noted that these images were different from those conveying depression, anxiety, and other native complex emotional pictures. For example, the mouths of individuals in the complex images conveyed happiness, while their eyes conveyed sadness. However, focusing on the mouth and eyes could induce a different emotional experience, which may affect the process of emotion regulation. This could be tested using the eye tracking technique in a future study. Second, we included college students as participants in this study. Future studies could validate the results with samples including individuals with schizophrenia, social anxiety disorder, bipolar disorders, and other clinical conditions.

## Conclusion

In conclusion, the present study revealed that when surprise was regulated, individuals’ surprise levels were lower and the N170 amplitudes were not influenced by emotion regulation, while the LPC amplitudes were smaller (compared with free viewing) after using expressive suppression and cognitive reappraisal (no difference), suggesting that the emotion regulation occurred at the late stage of facial expression processing, and the regulation effects of expressive suppression and cognitive reappraisal showed no significant difference.

## Methods

### Subjects

A total of twenty-two (12 women) healthy undergraduates from Chongqing University of Arts and Sciences were tested. All subjects range in age from 19 to 23, and their average age was 20.00 (SD = 1.10) years. All subjects reported no history of brain disease and mental illness, all of them were right-handed and reported normal or corrected to normal vision. All subjects were volunteering to participate in this test, and received 25 RMB for their participation. All participants provided written informed consent before the formal experiment, they were told their right to opt out at any time. The present study was approved by the Chongqing University of Arts and Sciences Human Research Institutional Review Board, which is in accordance with the Declaration of Helsinki (1991).

### Stimuli

Images of four types of facial expressions (neutral, happy, sad, and surprised, 30 of each type) and three Chinese words were used. Facial images were selected from the Chinese Facial Affective Picture System. In order to exclude the interference of the identity of the individuals on the pictures, the identity of the faces was matched during the selection of pictures, and only pictures of a single person were presented in each trial. Images of women and men were presented with the same frequency. All the images of men showed clean shaven male faces, while those of women included female faces with no earrings or neckerchiefs. Chinese words presented included “重评”, “抑制” and “观看”, which mean “cognitive reappraisal”, “expressive suppression”, and “freely view” (no strategy was used to regulate emotions), respectively. The font of the characters was Song Ti No. 32. Considering that the level of arousal impacts the regulatory effect of cognitive reappraisal^54,55^, the arousal and valence of emotions elicited by all images were indicated on a 9-point rating scale before the formal experiment. The complex emotional facial images (complex condition) were generated by FaceMorpher Lite 2.5, with the synthesis ratio being 1:1, using happy and sad as the original images, while the basic emotional facial images (basic condition) were neutral facial pictures. Additionally, the arousal and valence of the complex and basic images were evaluated by 20 individuals who did not participate in the formal experiment, and the results of independent-samples t-tests showed that the arousal level between complex (M ± SD, 2.712 ± 1.177) and basic (2.714 ± 1.176) images showed no significant difference, *t* = −0.007, *df* = 38, *p* = 0.995. Meanwhile, the valence of the two types of images (M ± SD, complex: 4.768 ± 0.621; basic: 4.776 ± 0.597) showed no significant difference either, *t* = −0.042, *df* = 38, *p* = 0.967. The physical properties of all pictures were controlled for, and the spatial frequency and brightness of all images (average gray scale value = 5.64 × 10^−5^) were the same. The brightness of a picture was obtained by averaging the gray scale values of all pixels of the picture, which was calculated using MATLAB 2014b. Both the complex and basic images were used to create different contexts, while the words were utilized to indicate which strategy the participants should adopt to regulate their surprise levels.

### Procedure

**Figure 4:**
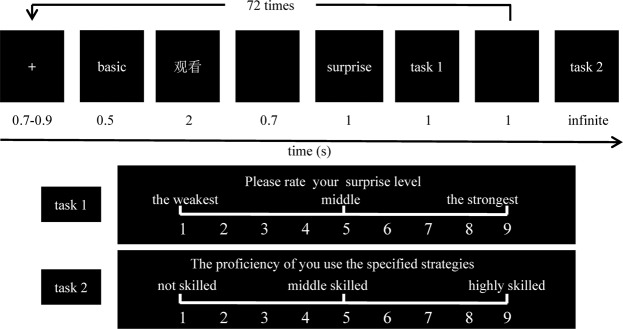
Flow chart of one block.

Stimuli presentation was controlled by the E-Prime 1.1 software (Psychology Software Tools Inc., Pittsburgh, PA, USA). The procedure involved five blocks, with each block including 72 trials, resulting in a total of 360 trials. As is shown in Fig. 4, each trial included the following order of presentation: a white “ + ” lasting for about 700 to 900 ms, followed by one of the contextual pictures (complex and basic images, 500 ms), and then one of the three Chinese words (“重评”, “抑制” and “观看”, 2000 ms), a blank screen (700 ms), a surprised facial expression (1000 ms), task 1 (up to 1000 ms), and a blank screen (1000 ms). Participants were asked to complete task 2 (with no time limits, and the stimulus did not disappear until participants replied) only in the last trial of each block. Both the complex and basic images were presented randomly and with equal probability, as were the three Chinese words. All the stimuli appeared in the center of the screen. Similar to previous studies^56–58^, both tasks were completed using a 9-point rating scale. In task 1, participants were asked to report their surprise level, after using the specified strategy to regulate their emotion; a response of “1” indicated “the weakest”, and “9” indicated “the strongest”. In task 2, participants were required to report the proficiency of their use of the three strategies. A response of “1” indicated “not skilled” and “9” indicated “highly skilled”. All participants were asked to reply using the number keys on the main keyboard. The two questions were presented in a fixed order. Participants were asked to complete the tasks as quickly and accurately as they could. In task 1, participants were required to answer within one second, while there was no time limit in task 2. Neither of the tasks had a standard answer, and no feedback was provided throughout the experiment.

The following details were emphasized before the formal experiment began. First, the implications of complex and basic images were explained. The emotions conveyed by both complex and basic images indicated that the person in the picture experienced an emotional event, and then experienced the corresponding emotions. The basic images implied that the character in the picture was undergoing a neutral emotional event, for example, he/she was sitting on a chair. The complex images implied that the person in the picture was undergoing an emotional uncertain (positive or negative) event, for example, he/she won (positive) or lost (negative) a large sum of money. To be specific, the mouth of the characters in these pictures conveyed happiness, but their eyes conveyed sadness. Thus, if one looked at the eyes of the character in the picture, he/she seemed to be sad because of losing money. Next, there was a causal relationship between the complex (basic) images and the surprised facial expression on the picture. In other words, the person in the image viewed the expression of the complex (basic) images, then he/she felt surprised.

Additionally, in accordance with previous studies^56^, the meaning of the terms “freely view”, “cognitive reappraisal”, and “expressive suppression” were as follows. When presented with “freely view”, the participants had to merely focus on the center of the screen; “cognitive reappraisal” meant that the participants had to believe that the character in the picture was not so surprised, i.e., lower the expectations regarding the level of surprise; “expressive suppression” meant that the participants were asked to try their best to inhibit their emotional reaction initiated by the images, and not show their emotions on their faces, such that others could not infer their inner feelings through their facial expressions. In order to deepen participants’ understanding of the three strategies, several examples were presented to them. To ensure that they truly understood the meaning of the three strategies, they were asked to illustrate those strategies through some examples before the practice test.

The present study was executed in a soundproof room, where participants sat in front of a computer (17-in screen, refresh rate = 60 Hz), the distance of participants’ eyes to the screen was 100 cm. In order to ensure that participants completely understood the experimental procedure, there were 12 practice trials before the formal experiment. The pictures used in the practice trials were different from the formal test. After participants fully understood the experimental procedure, they were asked to rest for a few minutes, and then began the formal experiment.

### Electroencephalogram (EEG) recording and analysis

Electrical activity data of the brain were recorded from 64 scalp sites using tin electrodes mounted on an elastic cap (Brain Product, Munich, Germany) according to the extended international 10–20 system, with FCz as the reference electrode. Vertical electrooculographies (EOGs) were measured via place an electrode 1 cm below the right eye, while the horizontal EOGs have not recorded, due to the design of the elastic cap. All impedance were kept below 5 kΩ. The EEG and EOG were amplified via a bandpass of 0.01–100 Hz, and sampled at a rate of 500 Hz. An off-line re-reference has been completed to obtain a global average. The EOGs were utilized to inspect artifacts induced by eye movement. During the off-line analysis, trials with EEGs and EOGs artifacts (amplitude of any electrode exceeded ± 80 μV) were excluded from averaging.

The EEG data were analyzed with the Brain Vision Analysis software 2.0 (Brain Product, Gilching, Germany). Behavioral and exported ERP data were analyzed with SPSS 16.0. ERP data were locked to different emotion regulation strategy. The ERP data were filtered through 0.01–30 Hz (24 dB/octave) bandpass filter. The average ERP epoch was 1200 ms, from 200 ms before and 1000 ms after the surprise faces onset. The segmentation was based on complex/basic images, as well as emotion regulation strategy (6 conditions in total). On the basis of vision inspection of the grand-average amplitudes and topographies, combining the measurement criteria of ERP references and waveforms^59^, the mean amplitudes of N170 and LPC have been analyzed. Based on previous studies and the topographical distribution of grand-average ERPs activity in present study, the average amplitudes of the N170 (165–185 ms) component were analyzed at P7, P8, PO7, PO8^38,60,61^, and the LPC (400–1000 ms) component were analyzed at P3, P4, Pz, PO3, PO4, and POz^31,32^. Mean amplitudes of the N170 and LPC have been analyzed through adopt a three-way repeated-measure analysis of variances (ANOVAs). Factors included in the analysis were context types (complex vs. basic), emotion regulation strategies (freely view, cognitive reappraisal and expressive suppression), and electrodes. All *p* values were corrected by utilization of the Greenhouse-Geisser correction.
